# Use of Robotic‐Arm Assisted Technique in Complex Primary Total Hip Arthroplasty

**DOI:** 10.1111/os.12659

**Published:** 2020-03-25

**Authors:** Wei Chai, Ren‐wen Guo, Ken Lee Puah, Seth Jerabek, Ji‐ying Chen, Pei‐fu Tang

**Affiliations:** ^1^ Department of Orthopaedic Surgery The General Hospital of the People's Liberation Army Beijing China; ^2^ Department of Orthopaedic Surgery Singapore General Hospital Singapore Singapore; ^3^ Department of Orthopedics Department of Orthopedics Hospital for Special Surgery New York, New York USA

**Keywords:** Ankylosing, Arthritis, Arthroplasty, Congenital, Hip, Hip dislocation, Replacement, Robotic surgical procedures, Spondylitis

## Abstract

**Background:**

There is a lack of data concerning the use of robotic devices in more complex total hip arthroplasty (THA) cases, such as hip dysplasia, ankylosing spondylolysis, and post‐traumatic arthritis.

**Case Presentation:**

This case study presented three cases in which the Mako robotic device was used to help accurately implement the surgical plan. The operations went smoothly. The position and angle of the acetabular shells were placed as planned without any complications related to the operation. Postoperative Harris Hip Scores were good in two patients and poor in the patient with ankylosing spondylitis. Robotic‐arm assisted surgery may be considered for complex THA cases in order to optimize the accuracy of the reconstruction, especially in the absence of conventional boney landmarks.

## Introduction

The Mako robotic‐arm assisted total hip arthroplasty (THA) was first introduced in 2010. The main aim of THA is reconstruction to provide a stable, pain free hip with high function. This is accomplished by restoring the center of rotation, leg length and offset with an acceptable component position. During the Mako robotic assisted THA, computer tomography (CT) based preoperative planning is used to accurately plan the hip replacement and the robotic arm is used to prepare the bone and place the components. Robotic‐arm assisted technology has been shown to recreate the head center, offset and leg length more accurately when compared to manual technique^1^, ^2^, ^3^, ^4^, ^5^. In a few published studies, robotic THA has been associated with improved patient reported outcomes, decreased dislocation rates, and high forgotten joint scores^1^, ^2^, ^6^, ^7^. However, these data have been on cohorts of patients with primary osteoarthritis of the hip. The aim of this study is to report on the use of robotics in hip replacement for more complex cases.

Hip dysplasia, ankylosing spondylolysis, and post‐traumatic arthritis are hip pathologies that often require THA^8^. Surgeons face a number of challenges while performing THA on these patients due to irregular joint anatomy. Dysplasia, Crowe III and IV in particular are challenging hip replacements given the distorted anatomy, high hip center, and lack of bone stock in and around the acetabulum^9^. Ankylosing spondylitis can lead to fused hips which are hard to dislocate and hard to reconstruct accurately^10^. Patients with post‐traumatic arthritis often have distorted anatomy, bone loss, and retained hardware hindering the reconstruction with THA^11^. There are also reports of increased complications with THA for these indications.

**Figure 1:**
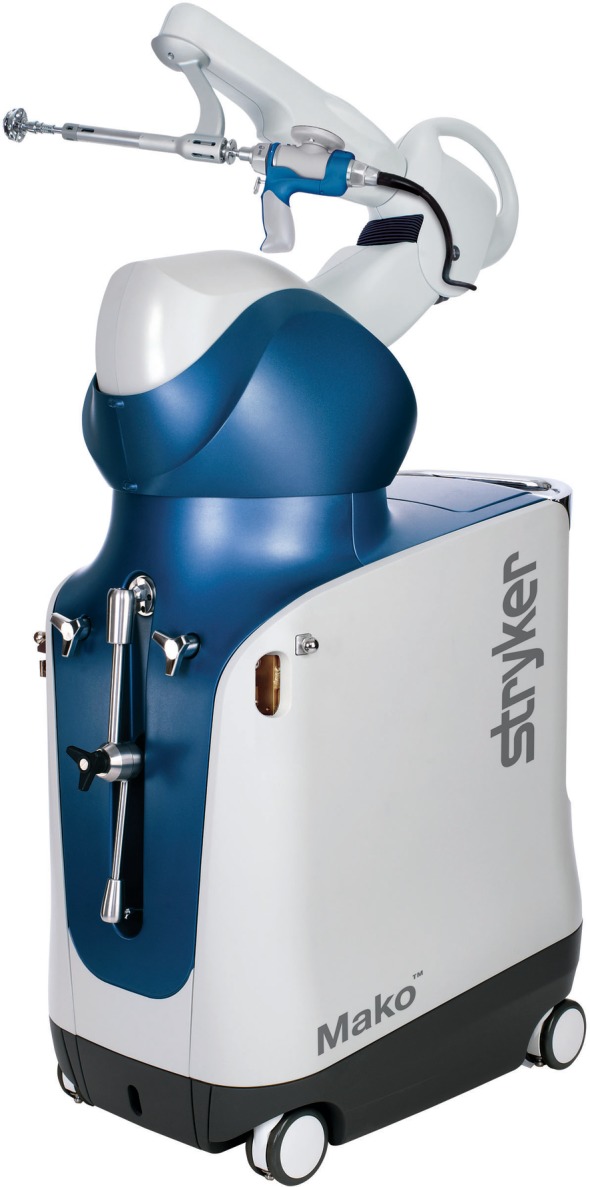
The Mako robotic arm for THA.

There is a paucity of clinical literature on patients suffering from hip dysplasia, ankylosing spondylitis, and post‐traumatic arthritis treated with THA using robotic‐arm assisted technique. The purpose of this case report was to demonstrate that highly complex THA cases can be performed with, and benefit from, the use of this technology to achieve excellent and predictable results. The model of the robot was RIO®, and the programming of the system was THA3.1 (Fig. 1). We used Express workflow in all the three cases.

## Case 1

### *History*

The patient was a 45‐year‐old male who had surgery for traumatic multiple bone fractures in 2016. He presented with persistent pain and limping after the trauma surgery and a decision was made to remove some of the plates and screws from both the femur and the tibia.

**Figure 2:**
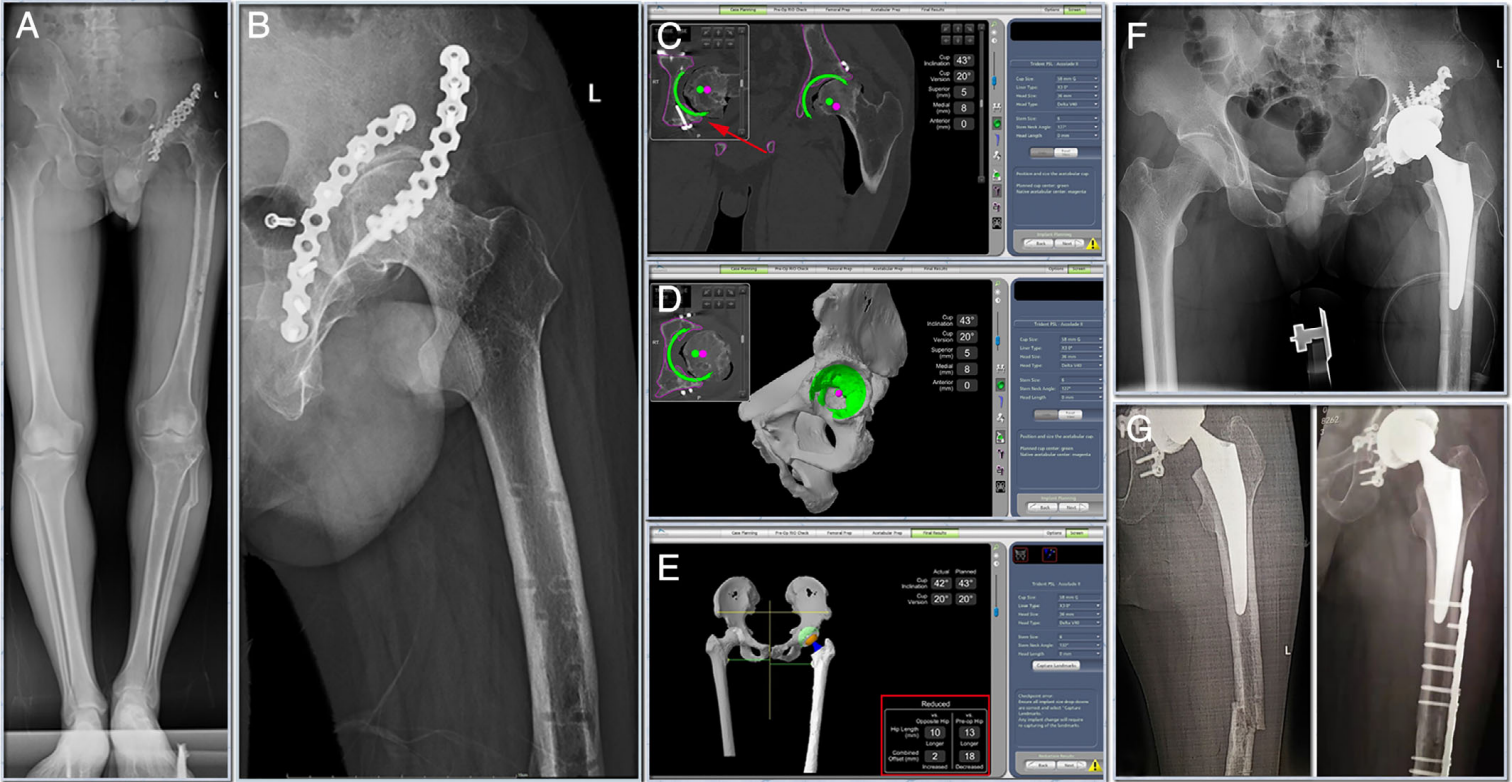
(A) & (B) Preoperative radiographs. The patient suffered from post‐traumatic arthritis of the left hip, with internal fixation on the anterior and posterior walls of the acetabulum. (C) & (D) CT based pre‐operative planning allowed to plan the cup away from the existing hardware (red arrow) in pelvis. (E) & (F) Robotic arm was used to execute that plan. And the leg length discrepancy was 10mm (red square). The simulation results of Mako system were the same as those of actual post‐operative X‐ray. (G) Patient's previous fracture site on femur recurred 1 month later, and then the fracture was held together by plate and screws.

In March 2018, however, no significant improvements were seen after hardware removal. On further examination, it was found that the left lower limb was 4 cm shorter than the right lower limb. Range of motion (ROM) test on left hip revealed that the patient was able to fully extend the left hip, but flexion was limited to only 20°. The patient's joint space was significantly narrowed. Osteophytes had developed around the articular surface and the patient reported tremendous pain and so a total hip replacement was planned (Fig. 2A,B).

### *Preoperative Planning and Intraoperative Execution*

With plates and screws on both anterior and posterior columns of the pelvis, it is critical to plan and place the cup away from them. CT‐based preoperative planning allowed to plan the cup away from the existing hardware in the pelvis (Fig. 2C,D). Once, the preoperative plan was agreed upon, the robotic arm was used to execute that plan intraoperatively.

Surgical approach was posterolateral approach, and the operation steps were consistent with the manual THA. First of all, specific bony markers need to be calibrated: (i) an EKG electrode was pasted on the lower edge of the patella (skin surface) with the knee flexed 90°; (ii) three drill bone pins were implanted on the anterior superior iliac spine (ASIS) of the operation side to firmly install the pelvic array; and (iii) acetabulum was exposed, marker screws were placed on the lateral side of the greater trochanter and above the acetabulum. A probe positioning EKG electrode and marker screws was used to let the robotic system locate the bony markers. Then, acetabulum registration was carried out, including 3 acetabulum align points, 32 registration points, and 8 verification points. Once the registration was completed, the three‐dimensional model established according to the preoperative CT scan aligned to the real bone. Then the acetabular reaming and acetabular shell impaction were under the guidance of the robotic arm. During the operation, the position and angle of acetabular shell could be adjusted according to the bone mass of acetabulum. Femoral preparation was done manually. In the process of trial reduction, with the knee flexed 90°, the probe was used to locate the marker screw of the great trochanter and the electrode of the patella, so that the system can feed back the data of leg length and offset. As the feedback data and stability test were satisfactory, the femoral prosthesis was installed to complete the operation.

We placed the cup in the desired location (43/20 planned *vs* 42/20 actual), and the leg length discrepancy was 10 mm (measured by the system) (Fig. 2E,F).The Trident cup and Accolade II stem (both Stryker Orthopedics, Mahwah, NJ, USA) were used in this case.

### *Postoperative Details*

Patient's previous fracture site on femur recurred 1 month after the total hip replacement. The complication was independent of the total hip replacement as it happened during rehabilitation and the patient reported no hip pain. Internal fixation was conducted. The fracture site was held together by plate and screws (Fig. 2G). Patient could walk without assistance during follow‐up at 3 months post‐operation, and the Harris Hip Score (HHS) 1 year after THA was 87 points.

## Case 2

### *History*

**Figure 3:**
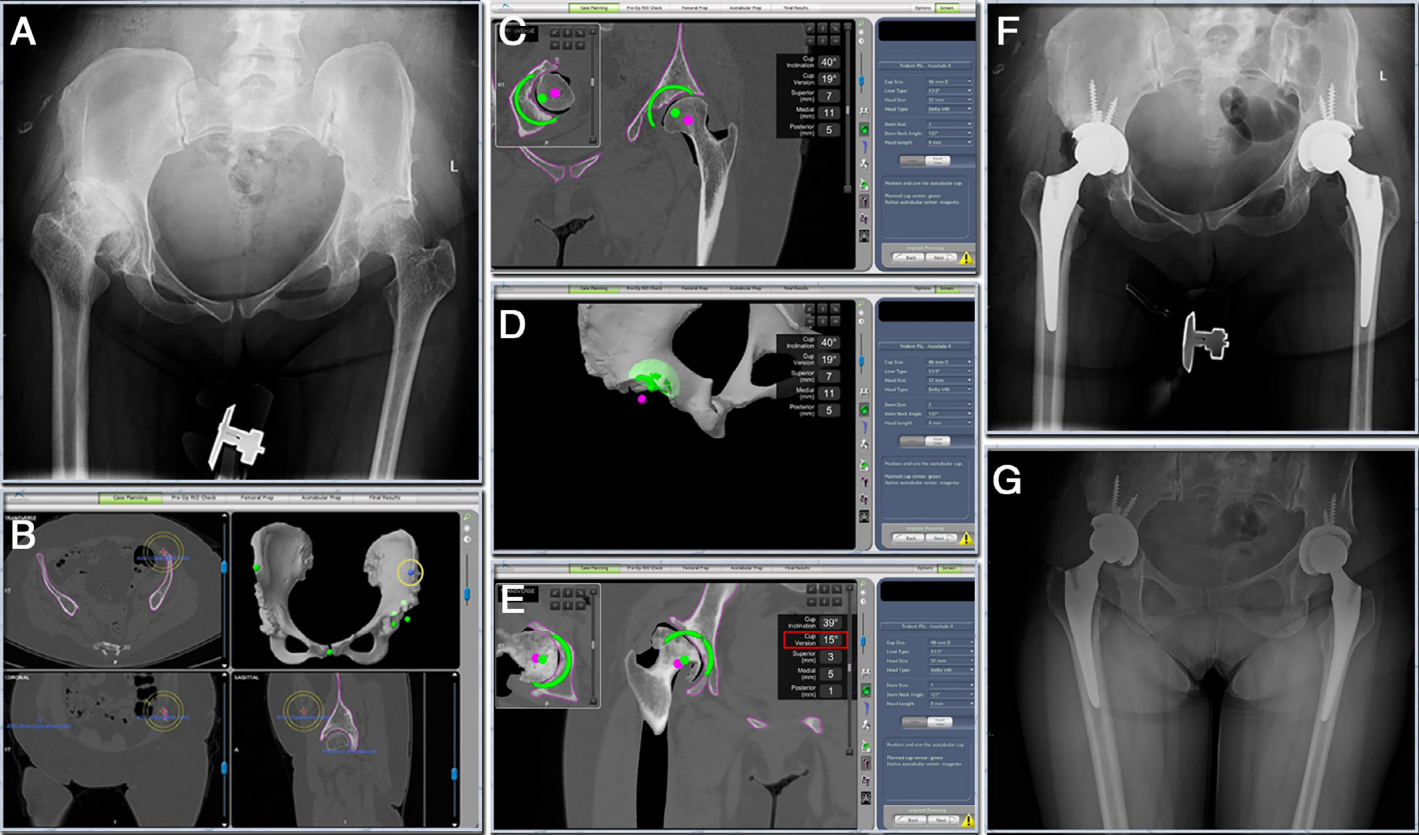
(A) Pre‐operative radiographs. The patient suffered from bilateral DDH. (B) & (C) & (D) & (E) CT based pre‐operative planning. Left ASIS was absent due to previous osteotomy (yellow circle). The position of the left ASIS reference point was carefully adjusted to make the pelvis axis as horizontal as possible. The cups were planned to reconstruct with the high hip center technique. The right cup anteversion was planned to be lower than usual (red square). (F) The post‐operative X‐rays showed that the robotic arm assisted THA had achieved the surgery plan. (G) Radiograph 3‐months post‐operative showed no change in the position of the prosthesis.

The patient was a 47‐year‐old female with developmental dysplasia of hip (DDH). The radiographic assessment determined that she had Crowe I on left hip and Crowe III on right hip. The patient had had a Salter innominate osteotomy done previously (Fig. 3A). During the visit, the patient presented with pain and limping. Physical examination revealed that both left and right hips could be fully extended and flexed.

### *Preoperative Planning and Intraoperative Execution*

Left ASIS was absent due to previous osteotomy, so the horizontal pelvic reference of the ASIS had to be accounted for and adjusted. By adjusting the position of the left ASIS reference point, the direction of the asymmetrical pelvis was adjusted to make the pelvis axis as horizontal as possible (Fig. 3B). Also, there was little bone stock for the array pins which required placing the pins at the iliac crest and fixed at the body of ilium bicortically. This case highlighted the benefits of 3D planning ahead of the surgery where there had to be a balance between optimizing the hip center of rotation and bony coverage/contact of the acetabular components. If the cups were planned at the native acetabulum, then there would have been defect superior to the cups. Given that the patient had bilateral hip dysplasia, the decision was made to proceed with a high hip center to maximize superior bone contact and to avoid femoral shortening osteotomies. The right femur was significantly anteverted in this patient, therefore the right cup anteversion was planned to be lower than usual (15°) (Fig. 3B–F).

### *Postoperative Details*

At 3‐months post‐operative the patient walked without assistance and reported no hip pain. The hips can be fully extended and flexed to 90°. The patient was satisfied with their leg lengths (Fig. 3G). The HHS at 1 year after THA was 83/86 points (left/right).

## Case 3

### *History*

**Figure 4:**
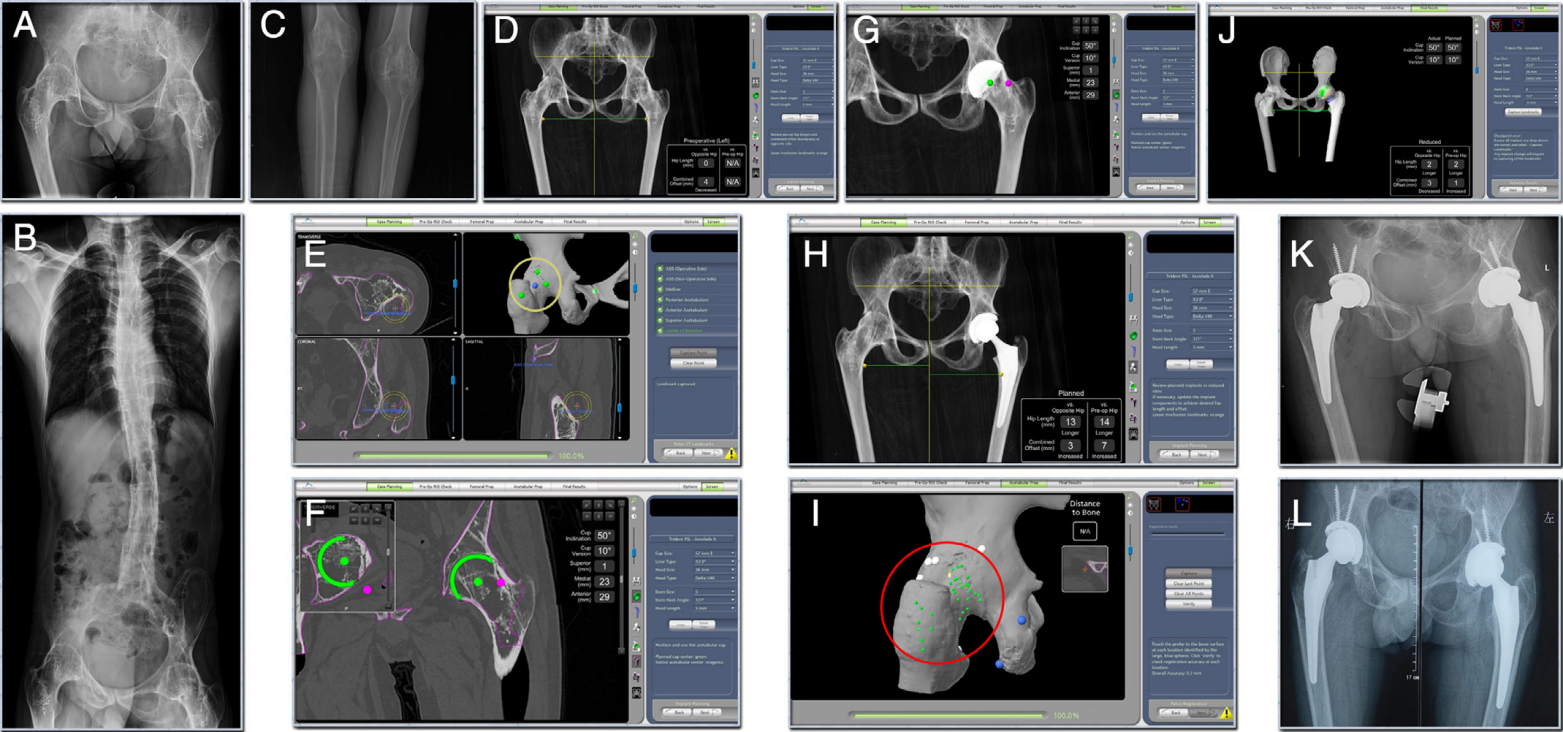
(A) & (B) & (C) Pre‐operative radiographs. Bilateral hips and knees have fused because of ankylosing spondylitis. (D) & (E) & (F) & (G) & (H) CT based pre‐op planning. The 3 acetabular align points that should had been set at the acetabulum were set at the great trochanter and acetabular rim (yellow circle). (I) & (J) & (K) Robotic arm was used to execute that plan. In the registration step of the operation, the circumference of the joint (great trochanter, femoral neck, acetabulum rim) was registered instead of the acetabulum (red circle). The robotic arm assisted THA had achieved the surgery plan. (l) Radiograph 3‐months post‐op**erative** showed no change in the position of the prosthesis.

The patient was a 38‐year‐old male with a history of ankylosing spondylitis. He had no prior hip surgeries. The patient presented with severe limping on both sides. The ROM on both left and right hip was zero secondary to bilateral hip ankyloses. The patient could hardly walk and moved by twisting his whole lower body to move forward or backward. A decision was made to perform bilateral THA on the patient (Fig. 4A–C).

### *Preoperative Planning and Intraoperative Execution*

Both left and right hips were completely fused, making dislocation impossible. As a result, in the preoperative plan it was decided to perform the segmentation of the femoral head and acetabulum as a single bone. The three acetabular align points that should had been set at the acetabulum were set at the great trochanter and acetabular rim (Fig. 4E). And in the registration step of the operation, the circumference of the joint (great trochanter, femoral neck, acetabulum rim) was registered instead of the acetabulum (Fig. 4I).This is an unusual process, and a significant deviation from standard protocol, which required specific surgeon approval; however, given the circumstances, it was deemed necessary to successfully plan and perform the surgery on this patient. Without being able to dislocate the hips, the exposure was very limited. Therefore the exposure was extended by about 1cm on the superior side and more superior soft tissue was stripped in order for a wider spread of registration, especially the superior area to the acetabulum rim. In such cases, extra caution needs to be taken to spread the registration points as far apart from each other as possible. This will ensure adequate registration despite the involvement of large surface area for matching. Other than the eight verification spheres, a manual check was performed to make sure the rotation was locked correctly. After we were certain the surface matching was successful, the hip was dislocated by osteotomy of the femoral neck *in situ* and then standard Mako procedure was followed (Fig. 4I–K).

### *Postoperative Details*

The patient underwent conventional bilateral TKA 15 days after the bilateral Mako THA. During follow up at 3 months post‐operation, both hips can be fully extended and flexed up 45° with assistance. Patient reported no pain and could walk with walking frame (Fig. 4L). The HHS 6 months after THA was 62 points (left and right).

## Discussion

Dysplasia, ankylosing spondylitis, and post‐traumatic arthritis present considerable challenges for performing THA. These cases have shown that robotic‐arm assisted surgery may offer significant benefits. The 3D planning allows the surgeon to precisely plan the surgery which is then executed by the robotic arm. CT based pre‐operative planning allowed the cup to be placed away from the existing hardware (Fig. 2C, red arrow) in the post‐traumatic arthritis case. In the dysplasia case, the plan was made to proceed with a high hip center to maximize superior bone contact and to avoid femoral shortening osteotomies. Registration presents challenges in these cases especially the ankylosing spondylolysis one. We have developed techniques for registration unreported before, which we have included here, that allowed us to achieve the accuracy required to restore the biomechanics of these hips to the best extent possible.

Several studies have examined the accuracy of the use of the robotic‐assisted device. Domb *et al*.^4^ performed a retrospective analysis of 50 patients treated with robotic‐assisted THA and compared them to a conventional control group of 50 patients and found 100% (50/50) of the robotic group were within the safe zone described by Lewinnek *et al*. compared with 80% (40/50) of the control group (*P* = 0.001). Similarly, Kanawade *et al*.^3^ performed a prospective study of 44 patients (48 hips) who underwent robotic‐assisted THA and found the 5° outlier rate of implant inclination/anteversion of the postoperative CT from preoperative plan were 88%/84%. In addition, Nodzo *et al*.^5^ conducted a prospective study of 20 patients on whom robotic‐assisted THA was performed and found inclination (40.4° ± 2.1°, 40.12° ± 3.0°; *R* ^2^ = 0.62; *P* < 0.001) and anteversion (23.2° ± 2.3°, 23.0° ± 2.4°; *R* ^2^ = 0.76; *P* < 0.001) and combined anteversion (32.5° ± 5.9°, 32.2° ± 6.4°; *R* ^2^ = 0.65; *P* < 0.001) were similar and correlated between intraoperative system reports and postoperative CT measurements. Gupta *et al*.^12^ performed a retrospective study in which 105 patients who underwent robotic‐assisted THA were divided into three groups by body mass index (BMI), and the study demonstrated there were no statistical differences in inclination (39.89° ± 3.02°, 39.72° ± 3.29°, 41.02° ± 2.27°, respectively, *P* = 0.43) and anteversion (16.78° ± 3.96°, 17.02° ± 3.60°, 16.73° ± 2.74°, respectively, *P* = 0.95) between the three groups (BMI < 30 kg/m^2^, BMI 30‐35 kg/m^2^, BMI > 35 kg/m^2^).

### *Conclusion*

We reported three challenging THA cases which were performed with the aid of robotic‐arm assistance. The operations went smoothly. The position and angle of the acetabular shells were placed as planned without any complications related to the operation. Postoperative HHS were good in two patients and poor in the patient with ankylosing spondylitis. The planning and accuracy of execution allowed us to give the patients excellent reconstruction of their hip joints which substantially improved their quality of life. Robotic‐arm assisted surgery may be considered for complex THA cases in order to optimize the accuracy of the reconstruction, especially in the absence of conventional boney landmarks.
